# Ground-Based Remote Sensing and Machine Learning for in Situ and Noninvasive Monitoring and Identification of Salts and Moisture in Historic Buildings

**DOI:** 10.1021/acs.analchem.4c05581

**Published:** 2025-02-25

**Authors:** Sotiria Kogou, Yu Li, C. S. Cheung, X. N. Han, Florence Liggins, Golnaz Shahtahmassebi, David Thickett, Haida Liang

**Affiliations:** † School of Science and Technology, Nottingham Trent University, Nottingham NG11 8NS, U.K.; ^‡^Institute for Cultural Heritage and History of Science Technology, University of Science and Technology Beijing, Beijing 100083, China; § English Heritage, Rangers House, Chesterfield Walk, London SE10 8QX, U.K.

## Abstract

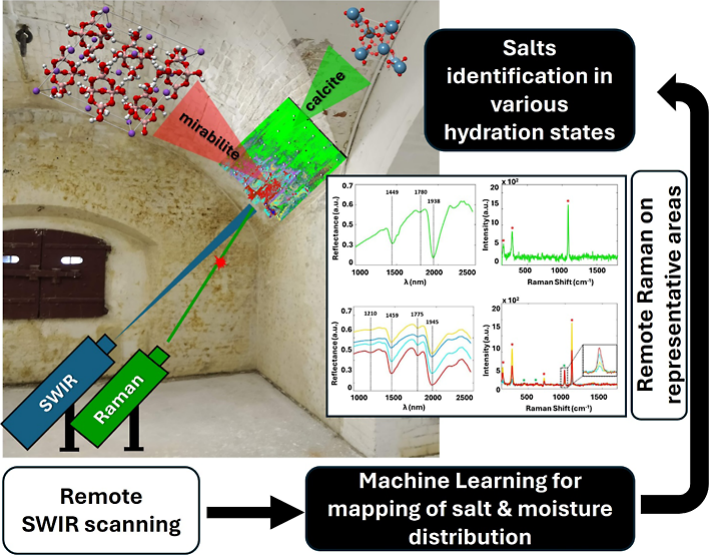

Historical buildings are prone to deterioration due to moisture and salt activity. Salt weathering affects the appearance of monuments, resulting in mechanical degradation. Many laboratory-based studies have been performed focusing on understanding salt formation in building materials and the resulting damage mechanisms. However, large-scale in situ monitoring is necessary to understand salt activity in realistic situations. Here, we present a novel methodology for in situ and noninvasive identification and monitoring of moisture and salts, following a complementary remote sensing approach. The study is based on ground-based remote short-wave infrared (SWIR) spectral imaging and remote Raman spectroscopy at stand-off distances of order 10 m. SWIR spectral imaging was used for scanning large wall surfaces at high resolutions (angular resolution of 45 μrad), which gave spatial distributions of moisture and salts in their various hydration states, visualized using an artificial neural-network based spectral clustering method. Remote Raman spectroscopy in each cluster area confirmed the identification of the salts.

## Introduction

Soluble salts in the pores of stones, bricks, and mortar are acknowledged weathering factors for historic buildings. Their crystallization and movement within the pores of building materials are factors for the propagation of various damaging phenomena.^1−5^ Depending on the environmental conditions, salts can crystallize either on the surface (efflorescence or compact crust) or within the porous body (subflorescence) of building structures. While efflorescence results in apparent alterations on a masonry’s surface, subflorescence is often related to more critical structural damage. Variations in the salt composition and hydration state result in differences in the nature and intensity of the corresponding weathering.^6−8^ The volume expansion of salts due to their hydration in the pores of building materials is probably a prime damaging factor for masonries. Sodium sulfate (Na_2_SO_4_), for example, undergoes an over 300% expansion when converting from its anhydrate state (thenardite) to the decahydrate state (mirabilite),^9^ making it one of the most dangerous salts. Fluctuations between various crystalline states accelerate the mechanical damage/material loss to the building materials.^10,11^ Therefore, identification and monitoring of moisture and salts are essential for determining the weathering condition of a historic building and the potential associated hazard.^12^

The traditional methods for identification of soluble salts rely on laboratory analysis of samples collected on-site, often combined with microclimate monitoring.^13−17^ These approaches present drawbacks when determining the hydration state of salts. Sample storage is challenging, as a slight temperature or relative humidity changes can trigger phase transitions and should be performed, such that it guarantees controlled environmental conditions. Moreover, laboratory analyses often require complicated and lengthy sample preparation procedures that can often result in phase changes and misinterpretations. To overcome the limitations of the sample-based analyses, recent studies introduced noninvasive and in situ measurements. Prieto-Taboada et al.^18^ presented the benefits of in situ Raman analysis for identifying different salts. Because localized measurements such as sample-based analysis or noninvasive spectroscopy at isolated points are not representative of the whole structure, a series of studies focused on in situ identification of salts and mapping their spatial distribution. Madariaga et al.^19^ suggested the combined use of portable Raman and hand-held X-ray fluorescence (XRF) spectroscopy to examine efflorescence formation. Given that both techniques provide point analysis, the investigation of the spatial distribution of specific salts was based on spatial interpolation of the XRF data.

Passive and active infrared thermography (IRT) analyses have been widely used for in situ monitoring of the spatial distribution of moisture in historic buildings. IRT has been combined with portable unilateral nuclear magnetic resonance (NMR) point analysis and gravimetric measurements for quantitative determination of the moisture content.^20^ However, IRT’s performance is subject to a range of parameters such as environmental conditions;^21^ gravimetric measurements are destructive and not always allowed. Di Tullio et al.^22^ suggested a method where the in-depth moisture monitoring provided by unilateral NMR is combined with evanescence field dielectrometry (EFD). EFD is a noninvasive technique that offers localized saline and moisture detection.^23,24^ Spatial distribution maps of moisture and salts were then obtained by interpolating the point analytical measurements from NMR and EFD. In this study, we propose a novel method for the identification and monitoring of moisture and salts using two ground-based remote sensing techniques in combination with machine learning methods for in situ monitoring of the spatial distribution of moisture and salts. The analytical techniques selected are remote SWIR (1000–2500 nm) spectral imaging and remote Raman spectroscopy (excitation wavelength at 780 nm) at distances on the order of 10 m for automated scanning of historic buildings. The ground-based remote SWIR spectral imaging provides high spatial and high spectral resolution imaging for the precision monitoring of salts and moisture. Water and salts have characteristic absorption bands in this spectral regime, and studies have shown that variations in the salts’ hydration state often translate to spectral differences.^25^ The large-scale SWIR spectral imaging data set is processed using an in-house developed machine learning method for unsupervised spectral grouping.^26^ Recently, we have also demonstrated the use of remote SWIR spectral imaging and machine learning for large-scale monitoring of bronze degradation.^27^ Here, we present how spectral clustering allows visualization of the spatial distribution of moisture and salts in their different hydration states. Remote Raman analysis within each SWIR cluster complements the investigation, offering a more specific identification of the salts. The effectiveness of the proposed method is illustrated via laboratory experiments and on-site analysis of the weathered interior of the caponiers of Fort Brockhurst, one of the Palmerston Forts located in Gosport, England.

## Results and Discussion

### Mock Experiments

**Figure 1:**
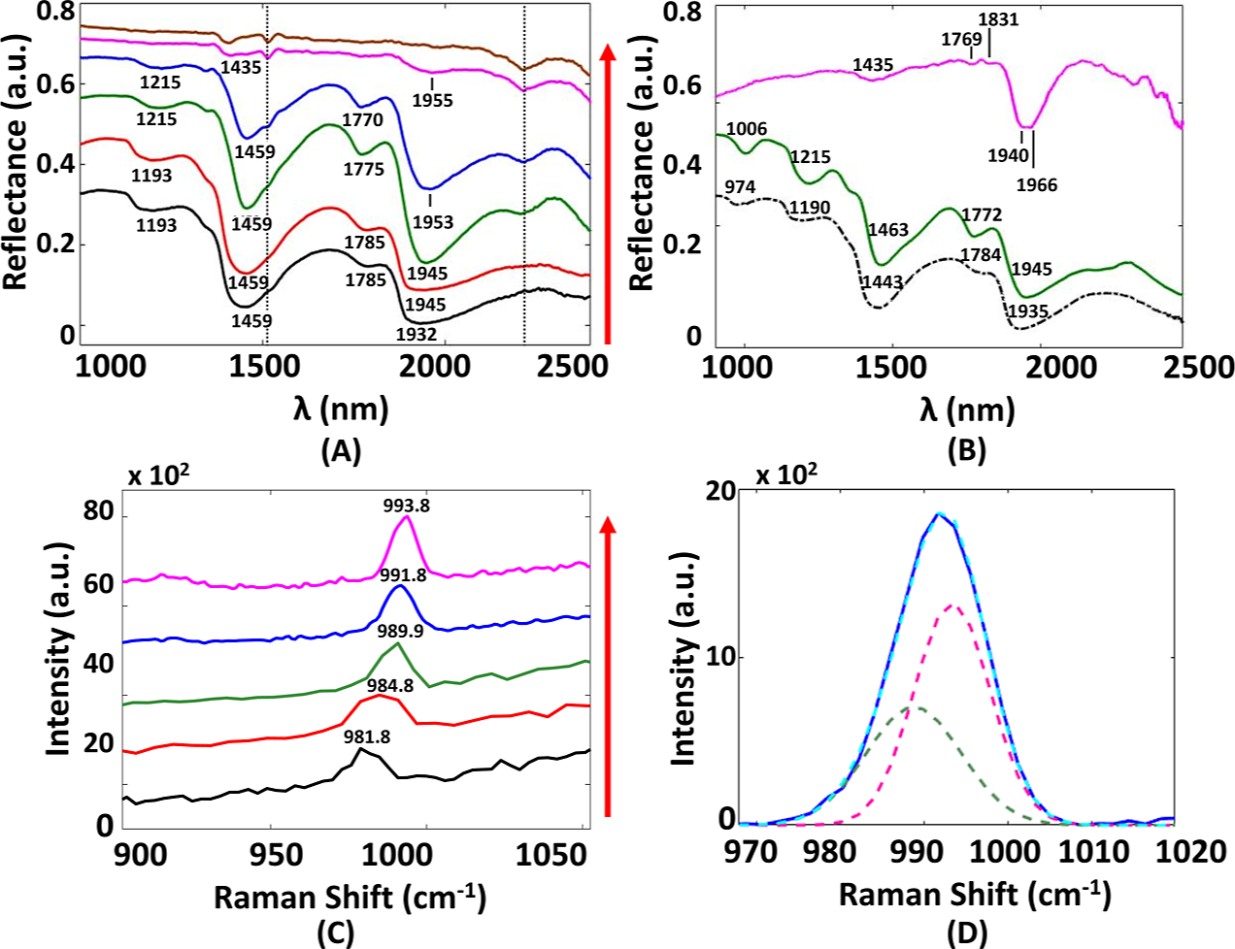
Laboratory investigation of Na_2_SO_4_ solution drying process (A) SWIR spectral evolution: Na_2_SO_4_ solution (black curve), heptahydrate (red curve), decahydrate/mirabilite (green curve), decahydrate/mirabilite and anhydrite/thenardite coexistence (blue curve), and thenardite coexistence (magenta curve). The reference spectrum of the sintered glass disk (brown curve) is plotted for comparison. The dashed vertical line indicates an instrumental artifact due to the order sorting filter. The spectra are vertically shifted for clarity and the order is from bottom to top as they appear during the drying process (red arrow on the right indicates the drying progress). (B) SWIR reference spectra of water (dashed black curve) collected in the lab, and mirabilite (green curve) and thenardite (magenta curve) as found in the UGSG library. (C) Raman spectral evolution of Na_2_SO_4_ solution drying process, focusing on the ν_1_ active vibrational mode of SO_4_^2–^; Na_2_SO_4_ solution (black curve), heptahydrate (red curve), decahydrate/mirabilite (green curve), phase between decahydrate/mirabilite, and anhydrite/thenardite (blue curve) and thenardite (magenta curve). The spectra are vertically shifted for clarity and the order is from bottom to top as they appear during the drying process (red arrow on the right indicates the drying progress). (D) ν_1_ line of the phase between decahydrate/mirabilite and anhydrite/thenardite (blue curve), along with the Voigt fit (dashed cyan curve), using as references the corresponding lines of the decahydrate/mirabilite (dashed green curve) and thenardite (dashed magenta curve).

The effectiveness of the combined remote sensing approach for monitoring and identifying salts in their various hydration states was evaluated by examining the drying process of Na_2_SO_4_ solution. Na_2_SO_4_ was selected as it is one of the most dangerous salts. The evolution of the SWIR spectra was examined based on shifts of the absorption bands related to the water molecules in the hydrated states and the presence of fluid inclusions and/or adsorbed water in the anhydrous state, thenardite. These shifts are indicative of various molecular interactions and provide insights into the hydration states of the salts. Specifically, the bands around 1200 nm are associated with a combination of the H–O–H bending fundamental and the first overtones of the O–H stretch. The features close to 1450 nm are linked to the first overtone of the O–H stretching fundamental. The bands around 1775 nm are related to combinations involving the fundamental H–O–H bend, the fundamental O–H stretch, and the low-frequency vibration modes of the structural water molecules. The strong features near 1900 nm are related to a combination of the O–H stretching and the H–O–H bending fundamentals.^28^ SWIR spectra collected during the drying process can be represented by five phases (Figure 1A). The spectra of these five phases were examined against the reference spectra of water, mirabilite, and thenardite (Figure 1B). The spectral feature of the Na_2_SO_4_ solution is similar to that of the pure water, with a slight shift to its absorption bands due to the presence of free ions. In the drying process, two stable forms of sodium sulfate were detected: decahydrate (mirabilite- Na_2_SO_4_. 10H_2_O) and anhydrite (thenardite- Na_2_SO_4_), respectively. The phase that appears between the solution and the mirabilite state consists of absorption bands from both of these phases. Similarly, the phase between mirabilite and thenardite seems to be a combination of these two stable hydration states.

The evolution of the corresponding Raman spectra confirmed the separation of the drying process in five phases, as indicated by the SWIR spectral imaging. The analysis was based on the observation of the line assigned to the ν_1_ active vibrational mode related to SO_4_^2–^ (Figure 1C). By closely examining the Raman spectra representing each phase, the identification of the solution, mirabilite, and thenardite states was confirmed. Moreover, the state between the solution and mirabilite was identified as heptahydrate (Na_2_SO_4_·7H_2_O). For the phase between mirabilite and thenardite, the line corresponding to the ν_1_ vibrational mode is slightly broader than the bands of these two stable states, with its position located between them. The deconvolution of this line into two peaks of Voigt profile, with central wavelengths and FWHM of the corresponding lines of the mirabilite and thenardite phase in Figure 1C is shown in Figure 1D, suggesting that mirabilite and thenardite coexist in this state.

Summarizing the mock experiments, we conclude that combined use of the remote SWIR spectral imaging and Raman systems could be used to detect and identify the various hydration states of Na_2_SO_4_. SWIR spectral imaging can offer time-efficient high spatial resolution (angular resolution of 45 μrad) large-scale scans. While this spectral regime does not necessarily provide identification with high specificity, it can be used for accurately discriminating the various states across the examined area. Raman spectroscopy, on the other hand, is a point analytical technique offering more specific identification of the salts.

### In situ Monitoring of Moisture and Na_2_SO_4_

**Figure 2:**
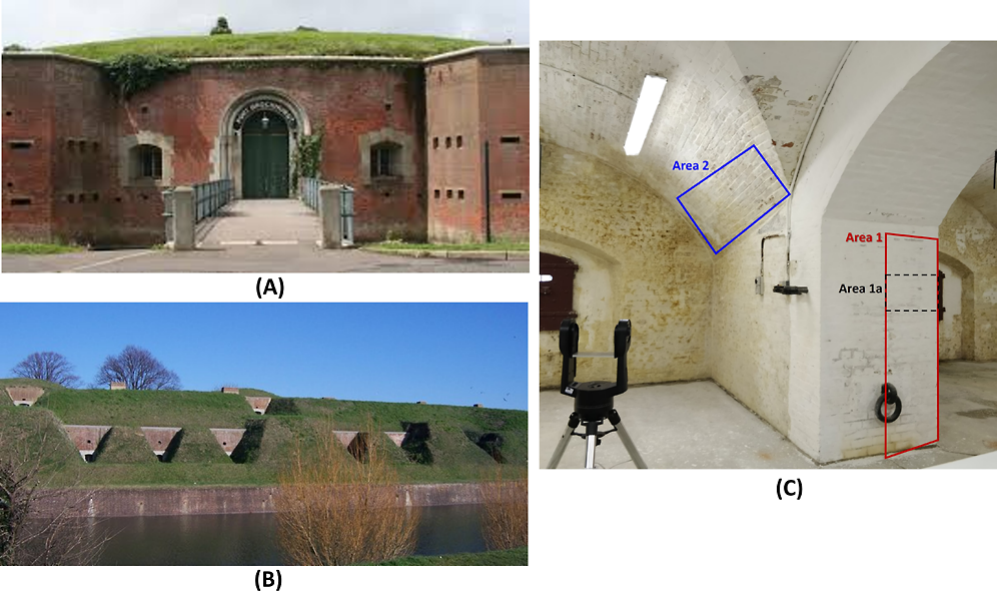
Fort Brockhurst along with the interior of part of the west-side caponiers (A) Fort Brockhurst, one of the Palmerston Forts, in Gosport, England. (B) Exterior of the caponiers located at the west side of the Fort. (C) Part of the interior of the caponiers, the colored boxes indicate the areas discussed in this study.

**Figure 3:**
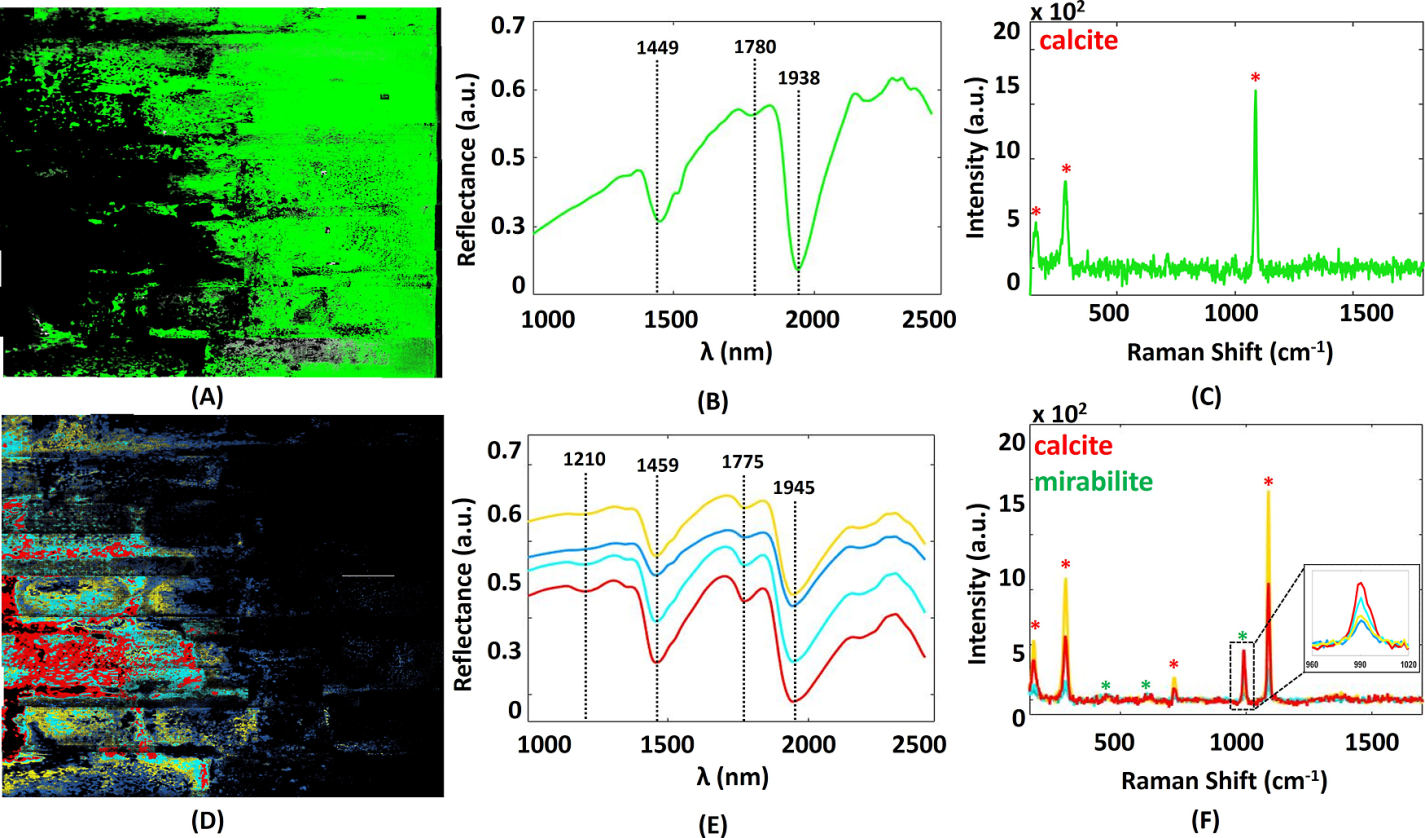
SWIR spectral clusters along with the corresponding Raman spectra. (A) SWIR cluster map of in bright green false color along with (B) corresponding SWIR mean cluster spectrum and (C) corresponding Raman spectrum; and (D) combined SWIR cluster maps in a yellow false color, cyan false color, red false color, and sky blue false color, along with (E) corresponding SWIR mean cluster spectra. The spectra are vertically shifted for clarity. (F) Corresponding Raman spectra (inset: zoomed in on the ν_1_ active vibrational mode of mirabilite).

A methodology that combines remote sensing with machine learning (description of the workflow presented in the Materials and Methods section) was followed for in situ monitoring and identification of the moisture and salts on the walls of Fort Brockhurst in South England (Figure 2A). The analysis was performed on the walls of the caponiers located on the west side of the fort. These caponiers are underground and by the moat’s side (Figure 2B), suffering from severe salt degradation. Remote SWIR spectral imaging was initially performed automatically on the walls of the west caponiers. A total of 6.5 m^2^ was scanned, translating to 1 × 10^8^ spectra. The large SWIR spectral imaging data set was reduced after processing with our machine-learning-based code for spectral grouping. Figure 3 illustrates the clustering results on the SWIR spectral imaging data set collected from a 1 m^2^ surface (Area 1a in Figure 2C), where a total of 32 million spectra was narrowed down to 11 clusters of distinct spectra.

The mean cluster spectrum of each SWIR cluster was examined in combination with their corresponding Raman spectrum to identify the salt species in their different hydration states and to examine the spatial distribution of moisture and salts. The areas covered by a solid whitewash layer at the edge of the wall are mainly represented by the cluster in Figure 3A, which has a mean cluster spectrum (Figure 3B) similar to the water spectrum shown in Figure 1B. The corresponding Raman spectrum (Figure 3C) detected calcite, which is associated with the whitewash material. Hardly any salts were detected in these areas. The areas further toward the center of the wall are represented by the cluster in Figure 3D. The corresponding mean cluster spectra (Figure 3E) share the same absorption bands, agreeing with the mirabilite state detected in the mock experiments (Figure 1A). The only difference between them is the overall spectral intensity. Raman spectroscopy (Figure 3F) collected in the areas corresponding to these clusters confirmed the identification of mirabilite. The whitewash material calcite was also detected in all areas.

**Figure 4:**
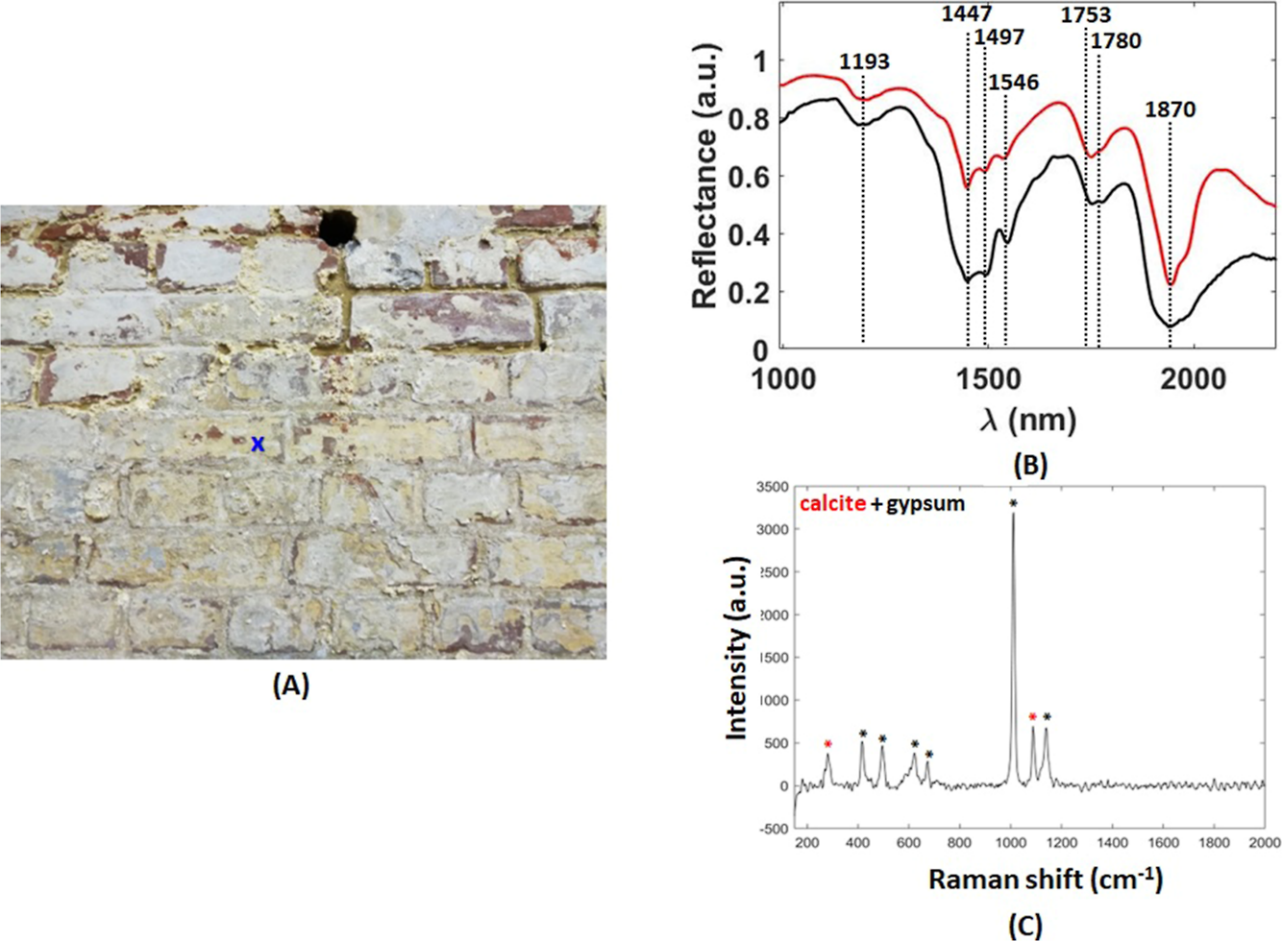
Salt examination following the complementary analytical approach on area 2 (see Figure 5B). (A) Zoomed-in RGB image of area 2 ((blue cross) indicates the area where the presented spectra data were collected from) and (B) spectral comparison between the corresponding SWIR spectrum (black curve) and the reference gypsum spectrum from the USGS online library (red curve), along with the (C) corresponding Raman spectrum.

Figure 4 shows that the same complementary analysis on area 2 (Figure 2C) gave another type of sulfate, gypsum (CaSO_4_. 2H_2_O). To understand the reason behind the formation of different sulfates on neighboring regions, the conservation history of the caponiers was examined in combination with their stratigraphy. According to restoration records, a waterproof membrane was placed above this area in the interface between the wall and the soil in the 1960s. However, this membrane failed over the years, letting water collect in the dips. Regarding stratigraphy, area 2 is part of the main 3-layered brick wall, located directly under these dips, while area 1 is part of the vertical side of the arches that are additions to the main wall. Therefore, the water content is expected to be higher in area 2 than in area 1, which means that the water flow in area 2 moved the sulfates from the soil to the whitewashed wall surface, resulting in the formation of gypsum.

This study introduces a novel methodology for in situ and noninvasive monitoring and identification of moisture and salts in historic buildings, combining remote sensing (SWIR spectral imaging and remote Raman spectroscopy at distances of order 10 m) with machine learning.

The combined use of these remote sensing techniques showed for the first time the potential for in situ monitoring and identification of sodium sulfate in all hydration states. Sodium sulfate was selected, as it is acknowledged to be one of the most dangerous salts. The study showed that the detection of gypsum is also possible. However, we aim to expand this investigation to all types of salts and examine more complex scenarios, i.e., the coexistence of various salts in various hydration states.

**Figure 5:**
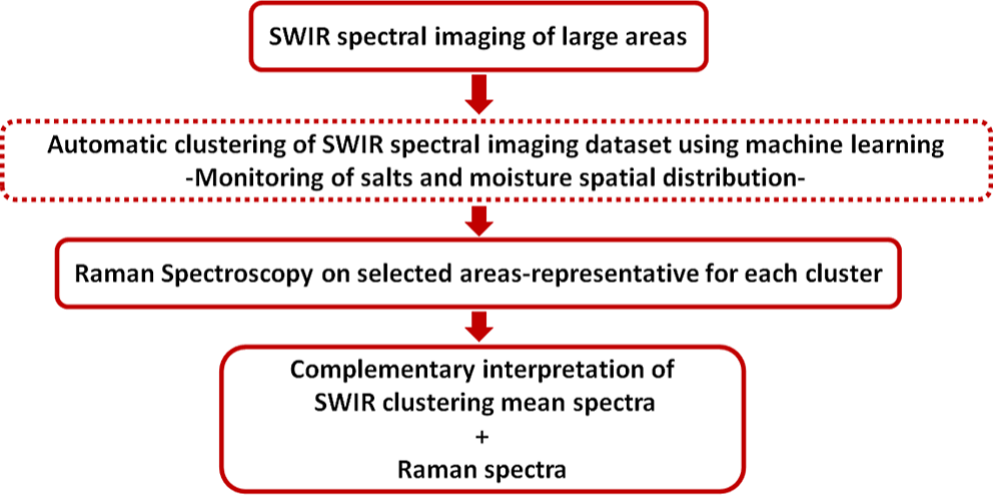
Novel methodology for the in situ and noninvasive monitoring and identification of moisture and salts on historic buildings. Diagram that describes the different stages of the new methodology that combines remote SWIR spectral imaging and Raman spectroscopy with artificial intelligence for the in situ monitoring and analysis of moisture and salts.

The advantage of the proposed methodology (Figure 5) is that it allows in situ large-scale noninvasive analysis, offering means of monitoring moisture and salts distribution across historic buildings with high specificity. It is particularly important to monitor the relative humidity and temperature when performing the analysis to ensure that the complementary analyses are performed under similar environmental conditions. Our method is a new tool for better understanding salt formation across a wall. For example, examining the wall of the arches in the caponiers of Fort Brockhurst reveals that moisture, but hardly any salts, was detected on the edges of the wall. In contrast, mirabilite was detected further toward the center of the wall with its concentration increasing closer to the center.

Another advantage of the proposed analytical approach lies in the noninvasive and noncontact nature of the in situ analysis enabling the revisiting of the site at different periods of the year to systematically monitor the hydration state in various environmental conditions.

## Materials and Methods

### Workflow

Figure 5 presents the workflow followed in this study. The first step in our methodology was time-efficient scanning of the large-scale wall surfaces using SWIR spectral imaging. The acquired large spectral imaging data set was then processed using an in-house-developed machine-learning method for clustering. Given that salts and water have characteristic absorption bands in the SWIR regime, the visualization of the clustering results in 2D maps allows for monitoring of their spatial distribution. The next step is the acquisition of Raman spectra from areas representative of each cluster. At the final stage, the complementary mean cluster SWIR spectra and the corresponding Raman spectra are interpreted in combination for the detailed identification of the salts in the various hydration states.

### Long Distance Stand-off Short-Wave Infrared (SWIR) Spectral Imaging

The Imaging and Sensing for Archaeology, Art History and Conservation (ISAAC) group adapted a HySpex SWIR-384 hyperspectral camera for high spatial resolution remote SWIR spectral imaging at distances of order 10 m in this study.^27^ It covers the spectral range between ∼950 and 2500 nm, with a spectral resolution of 5.5 nm and an angular resolution of ∼45 μrad which translates to 450 μm spatial resolution at 10 m. The field of view was ∼1°, while the integration time was 100 ms. The system was placed 7 m from the wall. The spectral imaging system attached on to a motorized pan/tilt stage, allowed automatic scanning of large surfaces (tens of square meters).

### Long Distance Stand-off Raman Spectroscopy System

A remote Raman spectroscopy system developed by the ISAAC group^28^ employs a continuous wave (CW) laser source for excitation at 780 nm, a telescope, and an Andor Shamrock spectrograph equipped with a high sensitivity Andor iDus CCD detector thermoelectrically cooled to −70 °C. The spectral resolution was 8 cm^–1^ over the spectral range of 130–3300 cm^−1^ with a 500 l/mm grating, while the spot size was 1 mm.

### Clustering Algorithm

The clustering method used in this study was in-house developed^26^ and was based on the self-organizing map (SOM) algorithm. The “kohonen” function from the built-in R stats package was used.^29^ This machine learning method enables unsupervised clustering as it learns directly from the input data set without needing a labeled reference database. SOM is an artificial neural network method with an input layer; in our case, the pixel-level SWIR spectra within a spectral image cube, mapped onto an output layer with nodes represented by the clusters. These two layers are connected by so-called weight vectors (mean cluster spectra). During the learning process, the output nodes (clusters) compete with each other for the input data (pixel-level spectra), with the corresponding weight vectors (mean cluster spectra) being constantly updated. The uniqueness of the final clusters is confirmed in the final stage of our clustering method by comparing their mean spectra with respect to the associated standard deviation (SD) per channel. The clusters remain separate if in at least one channel there is a difference that exceeds 2.5 SD.

### Mock Experiment Setup

To explore the potentials of the complementary use of remote SWIR spectral imaging and Raman spectroscopy for the identification of moisture and salts, we performed a series of lab experiments. In these experiments, the remote sensing systems were placed side-by-side and at a 3.5 m distance from the monitoring sample. The sample is a sintered glass disk 0 (porosity 30%, pore size: 160–250 μm) that was soaked in a Na_2_SO_4_ solution (20% concentration) for 30 min and then left to dry under ambient conditions. To monitor the various hydration states of Na_2_SO_4_, SWIR spectral imaging and Raman spectroscopic data were collected consecutively every 10 min.
